# Self-Supporting Flexible Paper-Based Electrode Reinforced by Gradient Network Structure

**DOI:** 10.3390/polym15061334

**Published:** 2023-03-07

**Authors:** Shaoran Kang, Zhijian Li, Jinbao Li, Hairu Wei, Yanbo Guo, Haiwen Li, Peng Yan, Haiwei Wu

**Affiliations:** 1 College of Bioresources Chemical and Materials Engineering, Shaanxi University of Science &Technology, Xi’an 710021, China; 2 Ningxia Shenyao Technology Co., Yinchuan 750004, China

**Keywords:** level three gradient network, reinforced, self-supporting paper-based electrode, flexibility, high capacity

## Abstract

At present, the self-supporting paper-based electrode has some problems, such as low mechanical strength and insufficient flexibility, which restrict its application in flexible electronics. In this paper, FWF is used as the skeleton fiber, and the contact area and the number of hydrogen bonds of the fiber are increased by grinding the fiber and adding nanofibers to bridge it, and a level three gradient enhanced skeleton support network structure is constructed, which effectively improves the mechanical strength and foldability of the paper-based electrodes. The tensile strength of FWF15-BNF5 paper-based electrode is 7.4 MPa, the elongation at break is increased to 3.7%, the electrode thickness is as low as 66 μm, the electrical conductivities is 5.6 S cm^−1^, and the contact angle to electrolyte as low as 45°, which has excellent electrolyte wettability, flexibility, and foldability. After three-layer superimposed rolling, the discharge areal capacity reached 3.3 mAh cm^−2^ and 2.9 mAh cm^−2^ at the rate of 0.1 C and 1.5 C, respectively, which was superior to the commercial LFP electrode, it had good cycle stability, and the areal capacity was 3.0 mAh cm^−2^ and 2.8 mAh cm^−2^ after 100 cycles at the rate of 0.3 C and 1.5 C.

## 1. Introduction

The development of flexible electrodes will greatly promote the application of flexible wearable devices and artificial intelligence devices [1]. Conventional electrode materials coated with metal current collectors are not foldable and the thickness of the active material coating is limited, which limits their application in the field of flexible energy storage equipment and the improvement of battery capacity [2,3]. Paper is an inherently flexible material [4]. The three-dimensional network structure of cellulose in the paper can not only provide mechanical support for matrix materials but also provide loading space for powder materials such as conductive agents and active substances to prepare three-dimensional network-like self-supporting electrodes [5,6]. This porous self-supporting structure is not only conducive to the improvement of the electrochemical performance of electrode materials [7] but also can increase the loading of active substances through proportional multiplication of electrode raw materials, i.e., to prepare thick electrodes [8]. Therefore, the preparation of paper-based electrode materials by papermaking technology provides a new way for the development of flexible high-load electrodes. At present, this kind of self-supporting paper-based electrode generally has some problems, such as low tensile strength, insufficient flexibility, and poor foldability [9,10,11]. The strength of paper is mainly determined by the strength of plant fibers and the bonding strength between fibers, including the contact area and the number of hydrogen bonds [4]. However, in the preparation process of the paper-based electrodes, the addition of a large number of conductive agents and active material particles blocked the contact between fibers and hindered the formation of hydrogen bonds between the fibers, resulting in insufficient flexibility, poor foldability, and low mechanical strength of the paper-based electrodes [12,13,14]. At present, the research on paper-based electrodes mainly focuses on the electrochemical activation modification of paper-based electrodes, such as enhancing their conductivity and energy density. Yue et al. [15] prepared porous flexible graphene paper electrodes by the vacuum filtration method with a specific capacitance of 468 F/g and a capacity retention of >81% after 10,000 charge/discharge cycles. Li et al. [16] used the capillary-assisted assembly method to coat graphene suspension on the surface of a nonwoven fabric and assembled a Graphene/MnO_2_ electrode material with good electrical conductivity and flexibility. The specific capacitance of this electrode material reached 138.8 m F/cm^2^, and the capacitance retention rate was 87.6% after 1000 charge/discharge cycles under a 180° bending angle. While the research on the enhancement modification and folding performance of paper-based electrodes is less.

**Figure 1:**
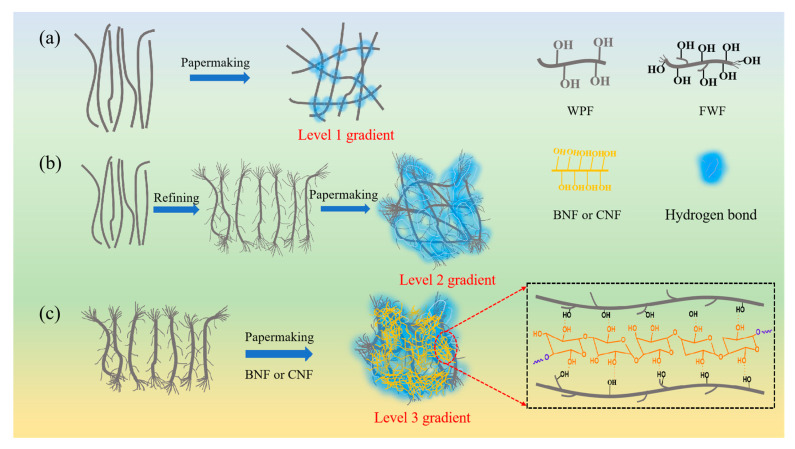
Fiber gradient reinforcement mechanism diagram. (**a**) Level one gradient skeleton support network formed by WPF fibers. (**b**) Level two gradient reinforced structure formed by WPF after pulping and papermaking. (**c**) Level three gradient-enhanced mesh structure formed by mixing FWF and BNF/CNF for papermaking.

To solve the above problems, a self-supporting paper-based electrode material with high mechanical strength, good folding performance, and excellent electrochemical performance were prepared by the paper-making process through the regulation of a fiber gradient network structure. The level one gradient mechanical support skeleton of electrode materials is formed by the network structure formed by staggered overlapping of plant fibers. However, the mechanical strength of this support skeleton is low because of the low content of plant fibers being blocked by conductive agents and active substances (Figure 1a). Therefore, by refining the plant fiber, the surface of the fiber is divided into fine fibers, and these fine fibers overlap with each other to form a level two gradient network (Figure 1b), which increases the hydrogen bonding sites, thus contributing to the improvement of the mechanical strength of the matrix material. Using the smaller size, the huge specific surface area, and abundant surface hydroxyl groups of nano-cellulose, a level three gradient network connection is formed between fiber filaments and plant fibers in a bridging way (Figure 1c). This level three gradient network greatly increases the hydrogen bonding sites between fibers in the matrix, which can effectively improve the mechanical strength and flexibility of paper-based electrodes [17]. In addition, this three-level network structure is more conducive to the envelope of micro-powder particles, reducing the shedding of conductive agents and active substances during folding deformation, thus improving the folding resistance of paper-based electrodes.

In this paper, paper-based electrodes with different fiber gradient structures were prepared by a paper-making process with coniferous wood fibers and nanocellulose fibers as the supporting skeleton, carbon nanotubes as the conducting agent, lithium iron phosphate as the active substance, and sodium dodecylbenzene sulfonate as the dispersant, after ultrasonic dispersion. Then, the flexibility of this self-supporting paper-based electrode in terms of capacity enhancement was verified by stacking multiple layers of paper-based electrodes.

## 2. Experiment Section

### 2.1. Materials

Wood pulp fiber (WPF, 90 wt%, coniferous bleached pulp) was purchased from North wood pulp and Timber Co., Ltd., Ottawa, ON, Canada. Cellulose Nanofilament (CNF, 1.0 wt%, Φ: 4~10 nm, L: 1~3 μm) and Bacterial nanocellulose fiber (BNF, 0.8 wt%, Φ: 50~100 nm, L: ~20 μm) were purchased from Guilin Qihong Technology Co., Ltd., Guilin, China. Carbon Nanotubes (MWCNT, L: ~50 μm) was purchased from Chengdu Institute of Organic Chemistry, Chinese Academy of Sciences, Chengdu, China. Lithium iron phosphate powder (LFP, LiFePO_4_, 98.00%) was purchased from Shanghai Maclean Biotechnology Co., Ltd., Shanghai, China. Sodium dodecylbenzene sulfonate (SDBS, C_12_H_25_SO_4_Na, 99.5%) was purchased from Tianjin Damao Chemical Reagent Factory, Tianjin, China. Electrolyte (LiTFSI/(DME:DOL)) was purchased from Suzhou Forsyth Technology Co., Ltd., Suzhou, China. PP diaphragm (Celgard2500, 25 μm) was purchased from Boliton Diaphragm Products Co., Ltd., Shanghai, China. The above raw materials are industrial products.

### 2.2. Preparation of LFP Paper-Based Electrode

A certain amount of WPF was added to 5 times of deionized water, and then the slurry was pulped by viscous pulping on a refiner to prepare filamented wood fiber (FWF), and the FWF with a beating degree of 65° RS.

A total of 15 mg of SDBS, 500 mg of WPF, 500 mg of MWCNT, and 2500 mg of LFP was added into deionized water with a volume of 1 L, and the digested mixed slurry was treated on a standard defibrillator for 5 min, and then added to a paper picker for papermaking and drying. Finally, the dried paper-based electrode sheet was rolled on a roller to the minimum thickness (the preparation process is shown in Figure 1) to obtain a paper-based electrode. It was marked as WPF20. The total amount of all fibers added was controlled to be 500 mg (absolute dry mass), and the types and proportions of fibers were changed according to the formula ratio in Table S1. LFP paper-based electrodes with different fiber components and contents were manufactured by the above process and were marked as FWF20, BNF20, FWF10-BNF10, FWF15-CNF5, FWF17-BNF3, and FWF19, according to the fiber addition ratio.

### 2.3. Assembly of Lithium Iron Phosphate Battery

**Figure 2:**
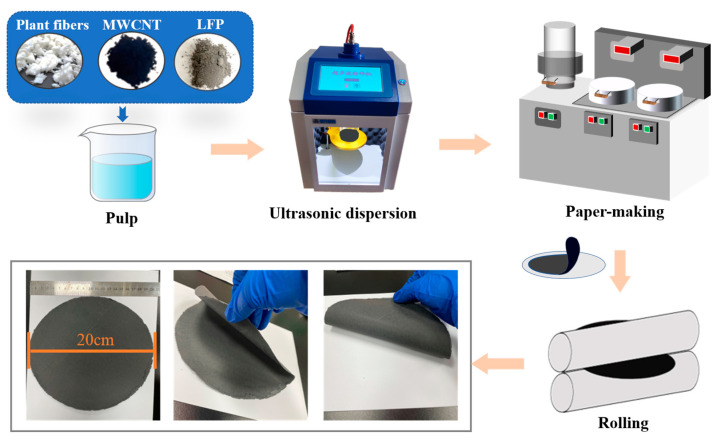
LFP paper electrodes preparation process.

The paper-based electrode prepared above was cut into a circular sample with a diameter of 12 mm by a cutter, and, after vacuum drying, the battery was assembled in a glove box filled with argon according to the stacking order of the button cell in Figure 2. Firstly, the electrode sample was placed on the positive electrode case stably, and 30 µL electrolyte was dripped, then the separator was covered on the papermaking electrode, then the lithium sheet (negative electrode) was placed on the separator, the gasket was added, and the negative electrode case was covered on it. Finally, the Ferrous lithium phosphate button cell was packaged with a sealing machine, and the assembled battery was allowed to stand for 12 h, and then its electrochemical performance was tested [18].

### 2.4. Characterization

The thickness was measured by an electronic spiral micrometer. The test of electrical conductivity was completed by a four-probe instrument (Four Probes Tech, Guangzhou, China, RTS-9). The sample was cut into strips of 2 cm in length and 1 cm in width, and the resistance at both ends of the long side was measured using a multimeter after folding 180° back and forth along the center line N times on the long side to measure the folding resistance of paper-based electrodes. The tensile strength and elongation at break were tested by a high and low temperature servo tension machine (Gotech, Shanghai, China, AI-7000NGD). The crystal structure was tested by an X-ray fluorescence spectrometer (XRD, Bruker, Shanghai, China, D8). The surface morphology was observed by field emission scanning electron microscope (SEM, Hitachi, Shanghai, China, S4800). The electrolyte wettability was tested by video optical contact angle measuring instrument (Dataphysics, Beijing, China, OCA20). The bending sensing volume resistance was tested by an automatic original analyzer (Tonghui, Guangzhou, China, TH2829A). The assembled button cell was tested on the electrochemical test system (Neware, Shenzhen, China, CT-4008).

## 3. Results and Discussions

Figure 2 shows the preparation of LFP paper electrodes. After following the pulping, paper-making, and rolling process, we were able to prepare large-size paper LFP paper electrodes with diameters of 20 cm. With different recipes, the mechanical strength, foldability, and also the preparation efficacy, which is mainly determined by the filtration time, all differ much. In order to achieve a high strength, high foldability, and high preparation efficacy of paper electrodes, and also check the effect of gradient network structure on all physical properties of paper electrodes, nine kinds of paper electrodes were carefully compared to illustrate the key point of making paper electrodes for potential application in flexible and high-capacity energy-storage devices.

### 3.1. Mechanical Properties and Thickness of the Paper-Based Electrodes

**Figure 3:**
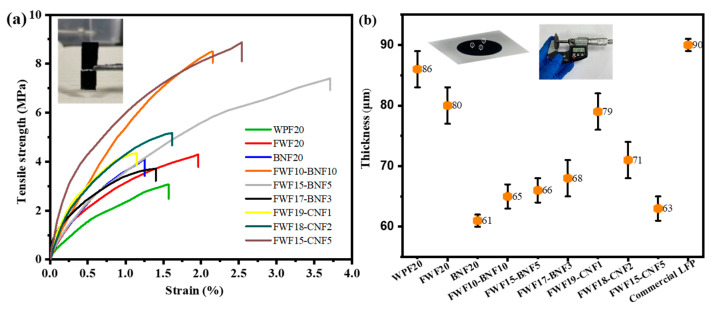
(**a**) Mechanical property curve of paper-based electrode; (**b**) Electrodes thickness.

Figure 3a is a stress curve of tensile strength and elongation at the break of the paper-based electrodes. By comparing and analyzing the mechanical properties of the paper-based electrode WPF20 supported by the level one gradient fiber skeleton, the paper-based electrode FWF20 reinforced by the level two gradient formed by fiber splitting and the paper-based electrode reinforced by the level three gradient formed by adding nano-cellulose fibers with different components and ratios, the mechanical enhancement effects of different gradient fiber structures on the paper-based electrode can be judged.

**Figure 4:**
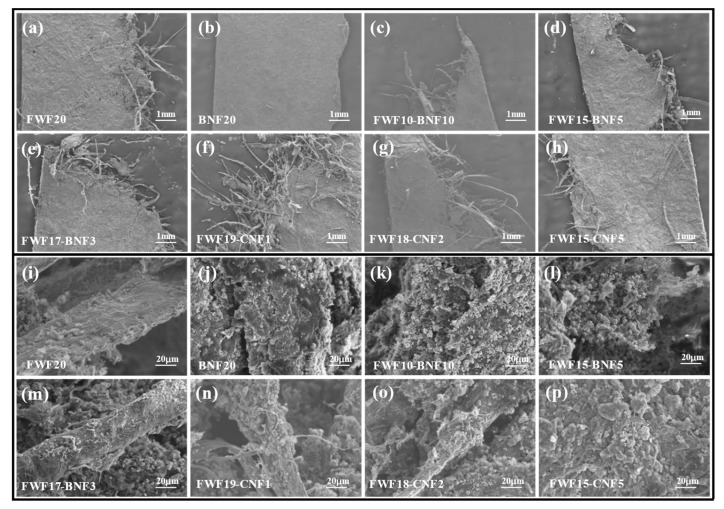
(**a**–**h**) are SEM images of paper-based electrodes with different nanofiber content at 50 times. (**i**–**p**) are SEM images of paper-based electrodes with different nanofiber content at 2 k times.

As shown in Figure 3a, the paper-based electrode prepared from WPF fibers without beating has the lowest tensile strength, only 3.1 MPa, and the elongation at break is 1.6%. The paper-based electrode with the level one gradient network structure has low mechanical strength because of the less -OH content on the fibers and the fact that the fibers are separated by conductive agents and active substances, resulting in fewer hydrogen bonds [19,20]. After refining WPF fiber, the fiber was divided into filaments and brushed, and the amount of -OH carried on the surface increased [21]. After preparing a paper-based electrode, the number of hydrogen bonds formed increased, and the bonding force between fibers was enhanced [22]. The tensile strength of the prepared WPF20 electrode material increased to 4.3 MPa, and the elongation at break increased to 1.9%. Although this level two gradient reinforced mesh structure can improve the mechanical strength of paper-based electrodes because of the increase in hydrogen bonds between fibers, the improvement of mechanical properties is limited because of the large size of FWF fibers, small contact area, and an insufficient number of hydrogen bonds [23,24,25]. To further improve the mechanical strength of paper-based electrodes, nano-cellulose was added to strengthen the combination between fibers in the form of bridging and filling, forming a level three gradient enhancement network. It can be seen from Figure 3a that the tensile strength of the material is improved after adding BNF or CNF, and the tensile strength of the material is gradually increased with the increase in the addition amount of BNF or CNF, in which the tensile strength of BNF20 is reduced because the fine nanofibers are blocked by larger LFP particles without the support of FWF fiber skeleton [26,27] (Figure 4j). In addition, because CNF has a smaller size than BNF, which is more conducive to filling the gaps between FWF fibers (Figure 4l,p), the tensile strength of CNF with the same quality is more obvious than that of BNF, and the tensile strength of FWF15-BNF5 is higher than that of FWF15-CNF5. Among all the paper-based electrodes, the tensile strength of FWF15-CNF5 is the largest, which is 8.9 MPa, and the elongation at break is 2.5%, while the elongation at break of FWF15-BNF5 is the largest, which is 3.7%, and the tensile strength is 7.4 MPa. The improvement of the elongation at break is beneficial to the toughness and foldability of the material.

As can be seen from Figure 3b, the thickness of all paper-based electrodes is smaller than that of commercial LFP electrodes, and with the increase in BNF or CNF addition, the thickness and error of paper-based electrodes become smaller and smaller, which is due to the level three gradient network structure formed by the addition of nano-cellulose [28], which strengthens the bonding force between fibers and makes the matrix denser (as can be confirmed by the surface topography of Figure 4a–h), thus reducing the thickness of the electrodes. The decrease in electrode thickness is beneficial to the improvement of volume capacity, while the decrease in errors shows that the uniformity of the electrode is improved, which is beneficial to the stability of electrochemical performance [29,30].

### 3.2. Conductivity of Paper-Based Electrode

Because the mechanical properties of the paper-based electrode (WPF20) with the level one gradient mesh structure are too poor to be used as a flexible material, the paper-based electrode with the level two gradient enhancement will be discussed directly.

**Figure 5:**
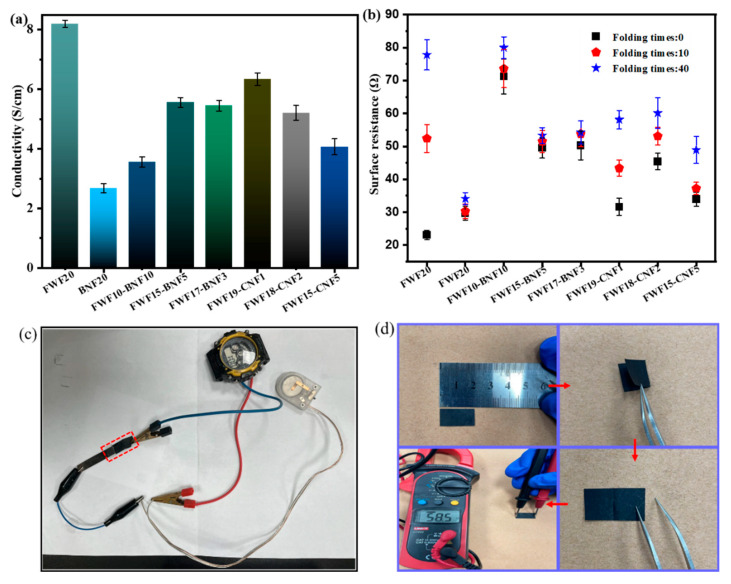
Physical properties of paper-based electrodes: (**a**) Electrical conductivity; (**b**) Surface resistance at different folding times; (**c**) Photograph of the watch powered by the battery assembled by FWF15-BNF5 and the conductive path connected by FWF15-BNF5; (**d**) Photograph of the paper-based electrode folding resistance test.

Because there is no metal current collector, the conductivity of a self-supporting paper-based electrode is one of the important indexes to evaluate the performance of the electrode [31], and the foldability of the paper-based electrodes is an important prerequisite to realize the flexible application [32]. As can be seen from Figure 5a, the conductivity of all LFP electrodes is between 2.7 and 8.2 S cm^−1^. When only FWF is added, the conductivity of FWF20 is the largest, which is 8.2 S cm^−1^. When nano-cellulose is added, the conductivity of LFP electrodes is decreased, which is due to the rich -OH surface of the nano-cellulose and the micro-nano size, which plays a better role in dispersing MWCNT [33]. The electrical conductivities of FWF15-BNF5 and FWF19-CNF1 are relatively good, which are 5.6 S cm^−1^ and 6.3 S cm^−1^, respectively. Figure 5c shows that the conductive path formed by the connection of FWF15-BNF5 can power the watch. As can be seen from Figure 5b, the surface resistance of FWF15-BNF5 and FWF17-BNF3 after being folded 10 and 40 times has little change compared with that before being folded, and the resistance base is small. This is because the addition of BNF increases the toughness of paper-based electrodes, and the electrodes are not prone to brittle fractures during the folding process [34], thus it has little influence on the formed MWCNT conductive paths. Figure 5d shows the folding resistance testing process of paper-based electrodes.

**Figure 6:**
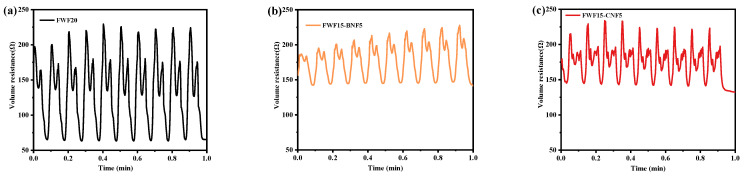

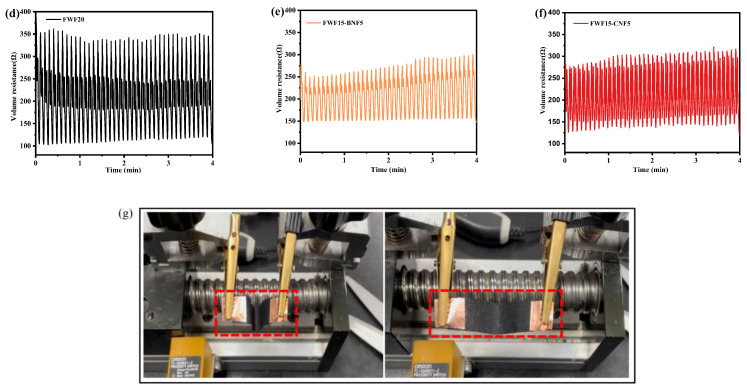
(**a**–**c**) Graph of sensing body resistance changes in paper-based electrodes bending-tensioning for one minute. (**d**–**f**) Graph of sensing body resistance changes in paper-based electrodes bending-tensioning for four minutes. (**g**) Photo of paper-based electrode bending-tension sensing body resistance test.

To further analyze the change in paper-based electrode resistance during bending deformation, the bending–stretching sensing test of the bulk resistance was carried out [35]. Figure 6a–c shows that the change range of body resistance of FWF15-BNF5 and FWF15-CNF5 is smaller than that of FWF20 within 1 min of bending–stretching, and the body resistance sensing signal of FWF15-BNF5 is more regular than that of FWF15-CNF5 during bending deformation, while the sensing signal of FWF15-CNF5 shows more miscellaneous peaks during 4 min of bending–stretching sensing test (Figure 6d–f). It shows that the lower body resistance fluctuation of FWF15-BNF5 in the bending deformation state is small and stable, which is more conducive to its application in the field of flexible electrode materials [36].

### 3.3. Contact Angle Analysis

**Figure 7:**
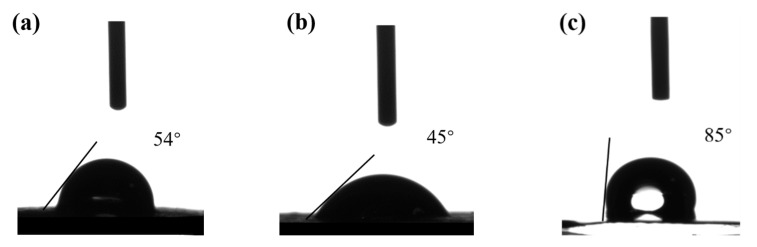
(**a**) is the contact angle between FWF15-CNF5 paper-based electrodes and electrolyte. (**b**) is the contact angle between FWF15-BNF5 paper-based electrodes and electrolyte. (**c**) is the contact angle between commercial LFP electrode and electrolyte.

Improving the wettability of electrode materials to the electrolyte is conducive to improving the electrochemical performance of materials. The better the performance of electrode wetting electrolytes, the better the specific capacity of materials, the higher the liquid retention capacity of batteries, and the smaller the polarization and attenuation during the battery cycle [37,38]. As can be seen from Figure 7, the contact angles between the two kinds of paper-based electrodes and the electrolyte are smaller than those of the commercial LFP electrode, and the contact angle of the FWF15-BNF5 electrode (45°) is smaller than that of the FWF15-CNF5 electrode (54°), indicating that the wettability of the electrolyte to the two kinds of paper-based electrodes is better than that of the commercial LFP electrode, and the paper-based electrode of FWF15-BNF5 has the best wettability. The matrix of commercial LFP electrode is made of metal, and the compaction density of active materials is high, which is not conducive to the infiltration of the electrolyte, while the self-supporting paper-based electrode has better infiltration of electrolyte because of the porosity of the material itself and the rich -OH on the fiber.

Based on the above performance evaluation, the FWF15-BNF5 paper-based electrode material has excellent conductivity, foldability, bending flexibility, wettability to electrolyte, and high mechanical strength, thus it can be used as the preferred electrode material in terms of physical properties.

### 3.4. Electrochemical Performance of Paper-Based Electrode

In terms of electrochemical performance, the FWF15-BNF5 paper-based electrode (areal capacity of 1.2 mAh cm^−2^ and LFP loading of 8 mg cm^−2^) is still lower than that of the commercial LFP electrode (areal capacity of 2.0 mAh cm^−2^ and LFP loading of 13 mg cm^−2^). However, compared with other traditional coated electrodes, the paper-based electrodes have the advantages that can continuously increase the LFP load through the proportional multiplication of papermaking raw materials on the one hand, and, on the other hand, it can increase the overall areal load per unit area and improve the unit areal capacity of the battery through multi-electrode rolling superposition [39]. In this scheme, the single electrode load of FWF15-BNF5 is kept unchanged, and three FWF15-BNF5 electrodes (marked as FWF15-BNF5*3) are rolled and stacked to assemble a lithium iron phosphate battery with a lithium-negative electrode, to verify the flexibility of multi-layer paper-based electrodes in the whole battery assembly process at one time.

**Figure 8:**
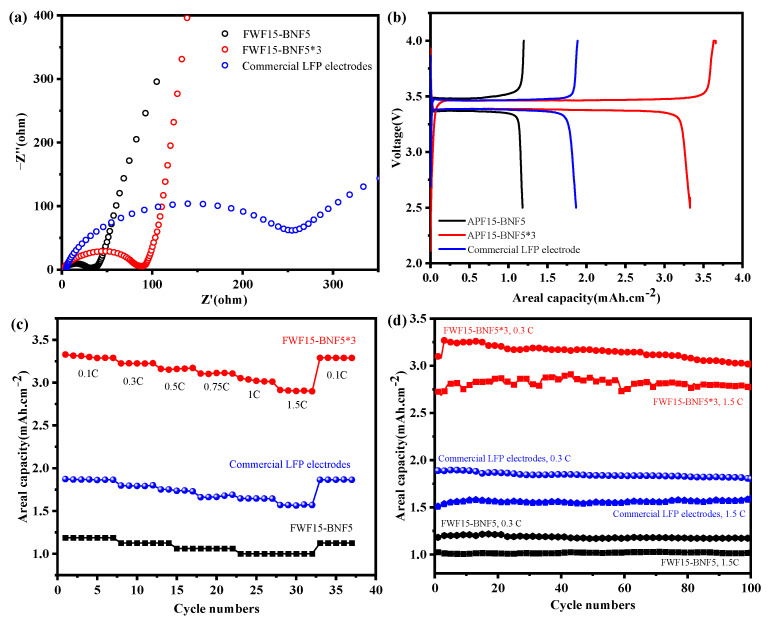
Electrochemical properties of paper-based electrodes. (**a**) EIS diagram of button cell assembled with each electrode material after three times activation. (**b**) First charge/discharge curve of button cell assembled with each electrode material. (**c**) Multiplicity curves of button cell assembled with each electrode material at different current densities. (**d**) Cycling performance of button cell assembled with each electrode material at different current densities.

The Nyquist curve of electrode material in Figure 8a shows that the semicircle of FWF15-BNF5 single electrode and FWF5-BNF5*3 three-layer superimposed electrode is smaller in the high-frequency region than that of commercial LFP electrode, but the slope in the low-frequency region is larger, indicating that this self-supporting paper-based electrode has smaller charge transfer internal resistance and higher ion diffusion efficiency, which is helpful to improve the electrochemical performance of the battery [40]. Figure 8b is the first charge–discharge curve of a button cell assembled with different electrode materials at a voltage range of 2.5–4.0 V and a current density of 0.22 mA cm^−2^ (0.1 C). It can be seen that the first discharge plateau voltage of paper-based electrode materials is stable at about 3.35 V, which is equivalent to that of commercial LFP electrodes, and the polarization is small. The discharge areal capacity of FWF15-BNF5 is 1.2 mAh cm^−2^. The discharge areal capacity of commercial LFP electrode is 1.9 mAh cm^−2^, while that of FWF15-BNF5*3 is 3.3 mAh cm^−2^, which is higher than that of commercial LFP electrode and 2.8 times that of FWF15-BNF5. It shows that the stacking process of the paper-based electrodes can improve the first charge and discharge areal capacity of the electrode, and the capacity loss after stacking is small. As can be seen from Figure 8c, with the increase in current density, the surface capacities of FWF15-BNF5, FWF15-BNF5*3 paper-based electrode, and commercial LFP electrode all decrease slightly and remain stable under different current densities. The discharge areal capacity of FWF15-BNF5*3 is better than that of commercial LFP electrode, and the paper-based electrode also shows good rate of performance, at a current density of 1.5 C (3.3 mA.cm^−2^). Figure 8d is a cyclic graph of a button cell assembled with different electrode materials after charging and discharging for 100 cycles at current densities of 0.3 C (0.66 mA cm^−2^) and 1.5 C (3.3 mA cm^−2^). It can be seen that the FWF15-BNF5 and the commercial LFP electrodes do not decay after 100 cycles, and the capacity retention rate is high, while the FWF15-BNF5*3 electrode slightly decays during the cycle. However, the overall areal capacity is still higher than that of the commercial LFP electrode, and the areal capacity is 3.0 mAh cm^−2^ and 2.8 mAh cm^−2^ after 100 cycles of charge and discharge at current densities of 0.3 C and 1.5 C, respectively.

In summary, the electrochemical analysis shows that this self-supporting paper-based electrode is more conducive to the transmission of charge and Li^+^ due to its porous structure, its impedance is smaller than that of commercial LFP electrode, and its rate and cycle performance are good. The superposition process has little effect on the electrochemical performance, and the areal capacity of the paper-based electrodes can be effectively improved by superposition.

## 4. Conclusions

Aiming at the problems of low mechanical strength, insufficient toughness, and poor foldability of the self-supporting paper-based electrodes, the mechanical strength and flexibility of paper-based electrodes were effectively improved through the regulation of a level three gradient network structure. The tensile strength of FWF15-BNF5 increased from 3.1 MPa of WPF20 to 7.4 MPa, and the elongation at break increased from 1.6% to 3.7%, which were increased by 241% and 236%, respectively. Moreover, the prepared paper-based electrode has both. In terms of electrochemical performance, this self-supporting paper-based electrode has lower impedance and higher ion transmission efficiency. What is more attractive is that the capacity of the FWF15-BNF5 electrode is doubled after three-layer superposition, and the first discharge areal capacity reaches 3.3 mAh cm^−2^, which is better than that of the commercial LFP electrode (1.9 mAh cm^−2^). Moreover, the superposition process has less impact on electrochemical properties and has excellent rate and cycle performance. The comprehensive performance is good, and it is suitable for large-scale industrialized and continuous production of papermaking technology.

Although the mechanical strength of paper-based electrodes was effectively improved in this study, there is still a gap compared with the strength of current commercial metal collector electrodes, and further improvement of their mechanical strength is needed to meet the needs of commercial applications. In addition, whether the paper-based electrodes prepared by this method meet the requirements of the production process of the paper industry on a large scale needs further validation and process adjustment.

## Data Availability

Not applicable.

## Supplementary Materials

The following supporting information can be downloaded at: https://www.mdpi.com/article/10.3390/polym15061334/s1, Figure S1. The XRD of paper-based electrodes and LFP. Table S1. Material addition ratio of LFP copying electrode. Table S2. List of acronyms.
