# Supplementary material for: Self-Supporting Flexible Paper-Based Electrode Reinforced by Gradient Network Structure
Source: Polymers (Basel). 2023 Mar 7;15(6):1334. doi: 10.3390/polym15061334 (PMC10059033; doi:10.3390/polym15061334)
Supplement: Supplementary file 1 [file polymers-15-01334-s001.zip › polymers-2229345-supplementary.pdf]

**Supporting Information**

# Self-supporting Flexible Paper-based Electrode Reinforced by Gradient Network Structure

**Shaoran Kang [1,2], Zhijian Li [1,*], Jinbao Li [1], Hairu Wei [1], Yanbo Guo [1], Haiwen Li [1], Peng Yan [2] and Haiwei Wu [1,*]**

1 College of Bioresources Chemical and Materials Engineering, Shaanxi University of Science &Technology, Xi'an 710021, China; ksr1988@126.com (S.K.); lijinbao@sust.edu.cn (J.L.); 15955175402@163.com (H.W.); guoyanbo180311@163.com (Y.G.); lhw3192710479@163.com (H.L.)
2 Ningxia Shenyao Technology Co.,Yinchuan 750004, China; yanpeng@nxsytc.com
* Correspondence: zjli@sust.edu.cn (Z.L.); haiweiwufly@163.com, wuhaiwei@sust.edu.cn (H.W.)

**Table S1 Material addition ratio of LFP copying electrode**

| Electrode name | Material added mass/mg | | | | | | |
|---|---|---|---|---|---|---|---|
| | WPF | FWF | BNF | CNF | CNT | LFP | SDBS |
| WPF20 | 500 | - | - | - | 500 | 2500 | 15 |
| FWF20 | - | 500 | - | - | 500 | 2500 | 15 |
| BNF20 | - | - | 500 | - | 500 | 2500 | 15 |
| FWF10-BNF10 | - | 250 | 250 | - | 500 | 2500 | 15 |
| FWF15-BNF5 | - | 375 | 125 | - | 500 | 2500 | 15 |
| FWF17-BNF3 | - | 425 | 75 | - | 500 | 2500 | 15 |
| FWF19-CNF1 | - | 475 | - | 25 | 500 | 2500 | 15 |
| FWF18-CNF2 | - | 450 | - | 50 | 500 | 2500 | 15 |
| FWF15-CNF5 | - | 375 | - | 125 | 500 | 2500 | 15 |

## X-ray diffraction (XRD) analysis

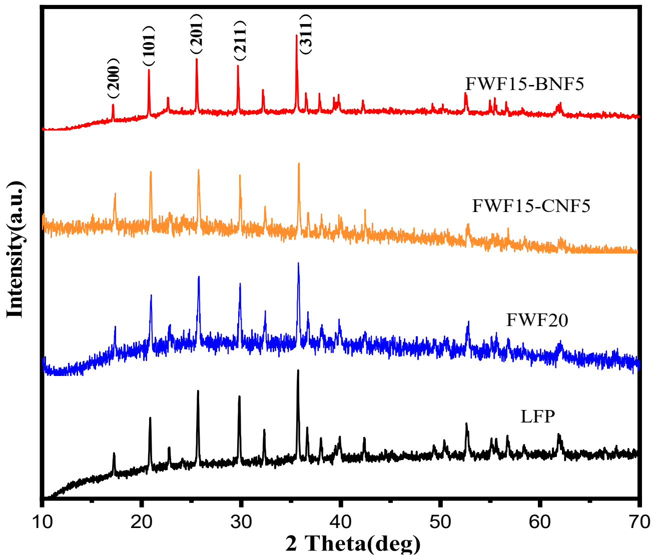


Figure S1. The XRD of paper-based electrodes and LFP

The optimized paper-based electrode materials of FWF15-BNF5, FWF15-CNF5, FWF20 and the active material LFP were compared and analyzed by XRD, to judge the influence of different fiber components and papermaking process on the crystal structure of the active material LFP. As can be seen from Figure S1, the XRD patterns of the paper-based electrode with BNF or CNF and the paper-based electrode without nano-cellulose unchanged and are consistent with those of LFP. Five characteristic peaks of LFP appear in all paper-based electrode materials, and the corresponding crystal plane indices are (200), (101), (201), (211), and (311) turn.

**Table S2 List of acronyms**

| List of acronyms | Acronym |
|---|---|
| Wood pulp fiber | WPF |
| Filamentied wood fiber | FWF |
| Lithium iron phosphate (LiFePO4) | LFP |
| Multi-walled carbon nanotubes | MWCNT |
| Sodium dodecylbenzene sulfonate($C_{12}H_{25}SO_4Na$) | SDBS |
| X-ray diffraction | XRD |
| Electrochemical impedance spectroscopy | EIS |
